# Ureteral Stents and Foley Catheters-Associated Urinary Tract Infections: The Role of Coatings and Materials in Infection Prevention

**DOI:** 10.3390/antibiotics3010087

**Published:** 2014-03-10

**Authors:** Joey Lo, Dirk Lange, Ben H. Chew

**Affiliations:** Department of Urologic Sciences, University of British Columbia, 2660 Oak Street, Vancouver, BC, V6H 3Z6, Canada; E-Mails: joey.lo@alumni.ubc.ca (J.L.); dirk.lange@ubc.ca (D.L.)

**Keywords:** urinary tract infection, catheter, ureteral stent

## Abstract

Urinary tract infections affect many patients, especially those who are admitted to hospital and receive a bladder catheter for drainage. Catheter associated urinary tract infections are some of the most common hospital infections and cost the health care system billions of dollars. Early removal is one of the mainstays of prevention as 100% of catheters become colonized. Patients with ureteral stents are also affected by infection and antibiotic therapy alone may not be the answer. We will review the current evidence on how to prevent infections of urinary biomaterials by using different coatings, new materials, and drug eluting technologies to decrease infection rates of ureteral stents and catheters.

## 1. Introduction

Catheter-associated urinary tract infections (CAUTI) are the most common source of hospital-acquired infections [1]. They account for 80% of all nosocomial infections worldwide, with approximately 450,000 cases in the United States alone annually [1]. As of 2008, treatment of these hospital-acquired infections is no longer eligible for reimbursement from the US Centres for Medicare and Medicaid Services, as they are considered to be preventable [2]. With direct treatment of CAUTIs amounting to over $350 million per year, it is crucial to prevent such infection [1]. Ureteral stents are hollow tubes used to facilitate the drainage of urine from the kidney to the bladder and are often used after treatment for kidney stones; since these are foreign bodies within the urinary tract system, they can also often lead to urinary tract infection. Current strategies to reduce ureteral stent infection have been mostly unsuccessful and it remains a clinical problem. For the remainder of this paper, stent and catheter infections will be considered together (CAUTI).

In order to develop effective mechanisms to decrease the incidence of CAUTI, the underlying sequence of events resulting in the development of infection need to be understood. When sterile urinary catheters and stents are inserted into the human body, components in urine, blood, or surrounding tissue, such as polysaccharides, ions, and glycoproteins, get deposited on the surface of the device [1,3] forming a urinary conditioning film. Considering that the conditioning film components have varying physical characteristics, their deposition alters the surface properties of the implants, allowing various planktonic bacteria to adhere to the surface via multiple mechanisms including electrostatic interactions and bacterial adhesins [4,5]. The initial interaction between bacteria and device surface is reversible as it is driven by weak hydrophobic and electrostatic forces [6], however, over time, the adherence becomes irreversible due to the binding of bacterial adhesins to their target molecules on the device surface as well as bacterial exopolysaccharide secretion, resulting in the formation of a biofilm. Biofilms are highly structured and actively growing bacterial communities that consist of multiple bacterial layers protected by a thick exopolysaccharide layer [6]. The presence of this thick protective layer combined with the fact that the phenotypes and metabolic functions of the embedded bacteria are modified, result in biofilms being significantly more resistant to antimicrobial drugs or disturbances than their planktonic counterparts [7] mostly due to the fact that antimicrobial agents cannot penetrate sufficiently through the exopolysaccharide layer towards the underlying bacteria as well as the strength with which it holds the community together. [8] As such, bacteria in a well-established biofilm have been shown to survive in antibiotic concentrations up to 1000-fold higher than the minimal inhibitory concentration for their planktonic counterparts [6,9]. As the biofilm becomes more developed, the expansion of the biofilm to “unpopulated” areas of the stents is facilitated by the detachment of bacterial cells from the biofilm followed by subsequent conversion back into planktonic, or free-swimming, state. Diffusion of these planktonic bacteria to non-colonized areas of the surface results in the initiation of new biofilm formation [10].

Device associated infections in urology are complicated by the fact that the majority of uropathogens are able to form these complex biofilm communities including both Gram positive and Gram negative bacteria, as well as yeast [9,11,12,13,14]. The most commonly isolated strains associated with uropathogenic biofilms are *Escherichia coli*, *Enterococcus faecalis*, and *Pseudomonas aeruginosa*, *E. faecalis* along with *Proteus mirabilis*, *Staphylococcus aureus* and *Candida tropicalis* are considered to be the strongest biofilm formers among uropathogens [15]. *P. mirabilis* biofilms are further complicated by the fact that it expresses urease, an enzyme capable of hydrolyzing urea up to 10× faster than the rate of other bacterial species. This process generates ammonia, which rapidly increases the alkalinity of urine significantly, creating an environment that promotes formation of hydroxyapatite and struvite crystals, resulting in a significant encrustation of the device surface [16]. Aside from promoting further bacterial adhesion and biofilm formation, these encrustations also block the catheter lumen often resulting in complete device failure [1,6].

While improved hygiene procedures as well as regular device replacement have helped decreased the incidence of CAUTI, it has not been prevented. As such it is crucial to develop strategies that will specifically inhibit the adhesion and growth of uropathogens. While the use of prophylactic antibiotics could be an effective way to kill potential intruders of the urinary tract prior to their adhesion to the device surface, it increases the development of further resistance and will not prevent the attachment and biofilm formation of uropathogens that are already resistant. As such approaches to prevent the initial bacterial attachment to surfaces need to be developed further, as this would prevent the bacteria from being retained in the urinary tract environment, being flushed back out by normal urinary flow.

To date, several approaches have been attempted: one of the most promising approaches involves the use of antimicrobial coatings on the surface of the urinary implants [1,3,17]. To date, various such coatings have been designed and tested for their anti-microbial activity, resulting in highly variable success rates in preventing CAUTI. These are discussed below.

## 2. Anti-Microbial Coatings to Inhibit Bacterial Growth and Adhesion

### 2.1. Antibiotic Coatings

The use of antibiotics can be traced back to decades ago, with the sulfonamide Prontosil being the first commercially available antibiotic developed in the 1930s [18]. Ever since then, several other classes of these bacteriostatic (preventing bacterial growth) or bactericidal (killing bacteria) compounds have been discovered, each targeting different bacterial functions or growth processes [19]. Initial approaches to coat device surfaces with antibiotics such as ciprofloxacin, gentamicin, norfloxacin, and nitrofurazone included dipping the implant into organic solvents containing antibiotics followed by the evaporation of the solvent and the deposition of the drug onto the device surface [20,21]. While this method was shown to be effective for short-term implants, it was complicated by uncontrolled release profiles of the drugs resulting in the elution of initial high local concentrations that may initially damage the cells followed by concentrations that are not inhibitory. Considering that this will not kill all of the bacteria effectively, it will result in subsequent infection that will be more difficult to eradicate due to the development of resistance [22]. As such the constant elution of at least the minimal inhibitory concentration of a given antibiotic is crucial for effective antibacterial activity.

Another more recent approach to apply antibiotics to implant surfaces is to incorporate the compounds into biodegradable coatings such that the drugs are released at the rate of the coating’s degradation [3]. Examples of such coatings are PVP (poly vinyl propylene), polyurethane, and calcium phosphate [3]. Similarly, enzyme-based drug elution technologies have been examined including the enzyme lipase B embedded in a polycaprolactone (PCL)-based coating containing the antibiotic gentamicin sulfate. In this elution mechanism the PCL serves as a substrate for lipase B, triggering the degradation of the coating with subsequent release of the antibiotic [3]. Despite the fact that this approach was shown to be effective for up to 16 days, it was abandoned due to problems with the biocompatibility of lipase B [3].

The combination of different antibiotics has also been shown to behave synergistically in preventing the adhesion and growth of uropathogens [23]. Using an *in vivo* rat model, Minardi *et al.* were able to demonstrate the efficacy of combining rifampin-soaked ureteral stents with intraperitoneal injection of tigecycline against the growth and biofilm formation of *E. faecalis* [23]. However, this method is only effective when both antibiotics are administered together, as resistance against rifampin is rapidly acquired by Gram positive bacteria when used alone [23].

### 2.2. Triclosan

Triclosan is an antimicrobial agent that has been used in consumer products for approximately 40 years [6]. It is a ubiquitous compound that affects both Gram positive and Gram negative bacteria affecting the stability of their cell walls—and it has been loaded into Foley catheters and ureteral stents [24,25,26]. Previous *in vivo* studies testing the efficacy of triclosan eluting stents in a rabbit model of UTI, demonstrated that triclosan-eluting stents significantly decreased the rate of *P. mirabilis* associated UTIs and bacterial load in comparison to non-eluting controls [26]. Moreover, Lange *et al*. were able to show triclosan-eluting stents to be effective in hindering bacterial adherence of uropathogenic *E. coli*, *Klebsiella pneumoniae*, and *S. aureus* [27]. In patients with long-term ureteral stents, the biofilm formation was too extensive and the amount of triclosan loaded into the stent was no match for the bacterial load that overwhelmed the ureteral stent within 3–6 months [28]. Although approved in many parts of the world, the triclosan eluting ureteral stent (Triumph^TM^, Boston Scientific Corporation, Marlborough, MA) was never FDA approved due to the potential concern that it would lead to further antibiotic resistance. Triclosan has never been shown to elicit any type of bacterial resistance—*in vitro* or in clinical studies [29]. In patients stented for less than 4 weeks, this stent did prove to relieve stent symptoms—giving credence to the theory that the anti-inflammatory effects of triclosan, rather than its antimicrobial effects, may be its most efficacious property in the urinary tract [30,31].

### 2.3. Silver

Silver is an effective broad-spectrum antimicrobial agent at low concentrations [2]. Implants composed from metallic silver have been shown to be inert prior to implantation, yet become highly reactive and ionize quickly once in contact with bodily fluids [1]. The reactive metal then generates silver ions capable of modifying bacterial cell walls and membranes, as well as inhibiting bacterial genome replication [1]. Although this type of metallic implant has been shown to be effective at preventing infections, patients are faced with significant morbidity including abdominal pain likely associated with the inflexible nature of the implant. Thus, alternatives such as silver-coated and silver-impregnated catheters have been proposed [12], however their effectiveness was found to be highly variable among studies [8]. Previous studies have found negative effects associated with the catheter materials, as silver-impregnated latex catheters were found to decrease bacterial adhesion more effectively than silver-impregnated silicone catheters [8]. Moreover, the activity of the silver particles was shown to be higher when particles are less than 10mm in size, shaped triangularly rather than spherically, and are present in oxidized rather than reduced forms [4,6]. These limitations significantly complicate the design of highly effective silver-based coatings. Potential problems associated with these coatings include argyria as a result of prolonged usage [3].

### 2.4. Hydrogel Coatings

Hydrogel is a hydrophilic, cross-linked polymer capable of absorbing large volumes of liquid [32] forming a thin layer of water on the coated device surface, preventing conditioning film formation by preventing the deposition of components including fibrinogen and platelets, that may facilitate bacterial adhesion [20]. In addition, due to the smooth and lubricious nature of hydrogel-coated catheters, these implants have been associated with less urethral irritation and inflammation [33]. In one study, hydrogel-coated silicone catheters resulted in 90% less *E. faecalis* adherence than uncoated catheters [33], although results from other studies were highly variable. In addition, a recent study by Elwood *et al*. has suggested that the presence of a conditioning film on catheter surfaces do not increase bacterial adhesion and colonization of urinary device surfaces compared to unconditioned controls [34].

### 2.5. Polyvinylpyrrolidone (PVP)

Similar to hydrogel, polyvinylpyrrolidone (PVP) is a hydrophilic, water-soluble polymer, capable of absorbing 40% of its weight with water [35,36]. Its excellent lubricant properties results in a soft, smooth and non-adhesive implant surface that facilitates device implantation, and was shown to reduce the adherence of *E. faecalis* and device encrustation *in vitro*, in comparison to uncoated polyurethane catheters [36].

### 2.6. Heparin Coating

Heparin is a highly sulfated glycosaminoglycan, often used as an anticoagulant with the highest negative charge density amongst all known biologic molecules [37]. Three different ways have been previously used to coat heparin on implant surfaces. These include physically adsorbing heparin, incorporating heparin into a polymer, and covalently binding heparin to implant surfaces via a spacer, with the last option being the most effective [37]. Covalently-bound heparin catheters have been shown to result in no detectable biofilm formation or encrustation for up to 6 weeks under clinical trials [38,39]. It is believed that the strong electronegativity of the coating repels microorganisms [37,38,39]. More recently, however, a study performed by Lange *et al.* studying bacterial adhesion to the surface of heparin-coated stents over a period of one week *in vitro* showed no decrease in bacterial adhesion to heparin-coated stents [27]. Although heparin coatings show great clinical performance in vascular catheters, the interaction of material and urine are not as beneficial as they are in blood making heparin a poor coating for urinary biomaterials.

### 2.7. Hyaluronic Acid Coating

Hyaluronic acid is a type of glycosaminoglycan [40]. It is an inhibitor of nucleation, growth, and aggregation of salts. Covalently bound hyaluronic acid catheters have been associated with increased hydration, decreased adsorption of proteins, and decreased bacterial adhesion [41]. While promising results were obtained in *in vitro* studies, the efficacy of hyaluronic acid coated stents in the clinical setting remains to be determined [41].

### 2.8. Gendine

Gendine is a novel antiseptic that contains Gentian Violet and chlorhexidine [6]. Catheters coated with this antiseptic have been shown to resist adherence of several multidrug-resistant bacteria in comparison to uncoated controls, as assessed by adhesion assays and scanning electron microscopy for visualizing biofilm formation [42]. When compared to silver hydrogel-coated catheters in a rabbit model, Gendine-coated urinary catheters were more effective at preventing bacterial colonization and urinary tract infections from *E. coli*, *P aeruginosa*, *K. pneumoniae*, and Candida species. Histopathologic examination showed no differences in inflammation between the silver coated catheters or the gendine antiseptic group.

### 2.9. Chitosan Coating

Chitosan is a natural cationic, biodegradable polysaccharide and a weak polyelectrolyte [10,43]. It is a non-toxic biopolymer obtained via chitin deacetylation, and has broad-spectrum activity against bacteria [10,43]. Although its exact mechanism is unknown, it is hypothesized to result in leaky cell membranes [10]. Due to its poorly soluble nature, chitosan has had limited capabilities for its use as a catheter coating. To overcome this limitation, water-soluble quaternised chitosan derivatives, known as hydroxypropyltrimethyl ammonium chloride chitosan, has been previously tested as a novel coating [43]. Recent studies have shown quaternised chitosan-loaded polymethylmethacrylate (PMMA) to be more effective at inhibiting biofilm formation of methicillin-resistant and non-resistant *S. epidermidis* and *S. aureus* than PMMA alone or gentamicin-loaded PMMA. Interestingly, these coatings were able to down-regulate the expression of genes encoding for enzymes responsible for biofilm biosynthesis, as demonstrated by real-time PCR [43].

To further strengthen the anti-adhesive and anti-bacterial capabilities of the chitosan coating, past researchers have constructed a multilayer film by assembling chitosan heparin in a layer-by-layer strategy onto aminolyzed poly(ethylene terephthalate) (PET) films [44]. This type of multilayer coating significantly decreased bacterial adhesion and the number of viable bacteria when co-incubated with *E. coli*. This multilayer coating demonstrated further antibacterial activity when silver nanoparticles were also incorporated [44].

### 2.10. Low-Energy Surface Acoustic Waves (SAW)

Low-energy acoustic waves transmitted directly to indwelling devices have been shown to inhibit bacterial adhesion to the implant surface by disrupting the formation of biofilms [45]. With a portable actuator generating piezoelectric vibrations between frequencies of 100 to 200 kHz, acoustic waves can be delivered to the surface of catheters [45]. Although there is no actual coating placed on the surface of implants, the waves cover the entire surface, generating a virtual vibrating coat [45]. This type of treatment has been shown to significantly inhibit biofilm formation of *E. coli*, *P. mirabilis*, and *Candida albicans* in comparison to non-vibrating controls [45]. The results have been confirmed using an *in vivo* rabbit model, where the treatment maintained sterility in urine for over one week in comparison to only 2 days in control animals [6,45]. However, to maintain the vibrating coating, elastic waves would need to be continuously delivered throughout the implantation process, which may be complicated by the fact that patients would be required to carry a portable actuator with them at times [45].

### 2.11. Salicylic Acid-Releasing Polyurethane Acrylate Polymers

Salicylic acid is a metabolite of aspirin, and has been known to have various effects on bacteria [46]. A recently studied salicylic-acid eluting coating involves ultraviolet-cured polyurethane acrylate polymer containing salicyl acrylate [1]. Under aqueous environments, the polymer hydrolyzes and releases salicylic acid, leaving the backbone intact. The rate of salicylic acid release is dependent upon the composition of the polymer, and the release of such acid was shown to inhibit biofilm formation of *S. epidermidis*, *Bacillus subtilis*, *E. coli*, *P. aeruginosa*, as well as *S. aureus* [1]. The effect was measured via a MBEC assay biofilm multi-peg growth system and bioluminescence monitoring, and the coated catheters were shown to be effective against biofilm formation of *E. coli in vitro* up to 5 days [1]. Although the mechanism is currently unknown, it is believed to hinder bacterial quorum sensing [1].

### 2.12. Antimicrobial Peptides Conjugated to Co-Polymer Brushes

Another more recent coating being studied in other areas of medicine includes antimicrobial peptides (AMPs) coatings [47]. These peptides are generally short, comprised of 10 to 50 residues of mainly lysine and arginine, making the peptide cationic [47,48]. Although the mechanism of action is not well understood, AMPs are believed to mainly disrupt the bacterial cell wall and cell membrane as well as affect DNA or RNA replication, protein synthesis, and many other bacterial processes [47]. Since AMPs are broad-spectrum and most likely target many processes rather than just one specific target, the likelihood of bacteria generating resistance against the peptides are relatively low, making them excellent antimicrobial agents [49]. In a recent study, antimicrobial peptides were coated on titanium implants via conjugation with hydrophilic polymer brushes [47]. These tethered peptides were capable of inhibiting bacterial growth both *in vitro* and *in vivo* using rat models [47]. During *in vivo* models, not only did the peptides show antibacterial effect, they also appeared to possess wound-healing effects [50]. Indeed, despite the promising results seen with AMPs, there are still caveats associated with the coatings. These include potential local toxicity, pH sensitivity, sensitization and allergy after repeated exposures, susceptibility to proteolysis, and the high cost of synthesis [49]. Despite this, the fact that AMPs can be easily modified to decrease these effects, their effectiveness as a catheter coating warrants further investigation as a novel approach to prevent bacterial adhesion and associated infection.

## 3. Conclusions

Although numerous antimicrobial coatings have been applied to ureteral stents and urinary catheters in an attempt to reduce CAUTI resulting from bacterial growth and adhesion on the medical devices, few of them of been shown to be fully effective. One of the major complications associated with antibiotic based coatings is the development of resistance. More novel antimicrobial agents, such as low-energy acoustic waves and antimicrobial peptides, are capable of avoiding this complication. However, in turn they may face other problems such as high cost of delivery or synthesis and potential toxicity. Recent studies have attempted to combine several antimicrobial agents into a single coating, such as the incorporation of several antibiotics into one coating or the multilayered heparin-chitosan film. However, further scientific and clinical studies are required before the ideal antimicrobial coating can be identified and introduced into clinical practice.
